# Induced Pluripotent Stem Cell-Derived Neural Stem Cell Therapy Enhances Recovery in an Ischemic Stroke Pig Model

**DOI:** 10.1038/s41598-017-10406-x

**Published:** 2017-08-30

**Authors:** Emily W. Baker, Simon R. Platt, Vivian W. Lau, Harrison E. Grace, Shannon P. Holmes, Liya Wang, Kylee Jo Duberstein, Elizabeth W. Howerth, Holly A. Kinder, Steve L. Stice, David C. Hess, Hui Mao, Franklin D. West

**Affiliations:** 1 Regenerative Bioscience Center, University of Georgia, Athens, GA 30602 USA; 2 Department of Animal and Dairy Science, University of Georgia, Athens, GA 30602 USA; 3 Department of Small Animal Medicine and Surgery, University of Georgia, Athens, GA 30602 USA; 4 Department of Veterinary Biosciences & Diagnostic Imaging, University of Georgia, Athens, GA 30602 USA; 5 Department of Radiology and Imaging Sciences, Emory University, Atlanta, GA 30329 USA; 6 Department of Pathology, University of Georgia, Athens, GA 30602 USA; 7 Department of Neurology, Augusta University, Augusta, GA 30912 USA

## Abstract

Induced pluripotent stem cell-derived neural stem cells (iNSCs) have significant potential as an autologous, multifunctional cell therapy for stroke, which is the primary cause of long term disability in the United States and the second leading cause of death worldwide. Here we show that iNSC transplantation improves recovery through neuroprotective, regenerative, and cell replacement mechanisms in a novel ischemic pig stroke model. Longitudinal multiparametric magnetic resonance imaging (MRI) following iNSC therapy demonstrated reduced changes in white matter integrity, cerebral blood perfusion, and brain metabolism in the infarcted tissue. The observed tissue level recovery strongly correlated with decreased immune response, enhanced neuronal protection, and increased neurogenesis. iNSCs differentiated into neurons and oligodendrocytes with indication of long term integration. The robust recovery response to iNSC therapy in a translational pig stroke model with increased predictive potential strongly supports that iNSCs may be the critically needed therapeutic for human stroke patients.

## Introduction

Stroke is the leading cause of long-term disability in the United States and the second leading cause of death worldwide^1^. Due to the epidemic magnitude of stroke, hundreds of clinical trials have been performed to assess therapeutics that mitigate the sequela of stroke injury^2^. Unfortunately, these treatments have all failed in clinical trials with the exception of tissue plasminogen activator (tPA), the only Food and Drug Administration (FDA) approved agent for treating stroke. Despite the benefits of tPA, <5% of patients receive this treatment due to its narrow therapeutic window and its effectiveness in only ischemic stroke^1, 3, 4^. The mechanism of tPA and the majority of other tested therapeutics focus on limiting ischemic damage by promoting reperfusion of brain tissue or acting as neuroprotectants. However, their functions have no direct restorative or regenerative action that would enhance replacement of brain tissue leading to improved functional recovery^4, 5^. An assessment of the litany of failed clinical trials by the Stem Cell Emerging Paradigm in Stroke (STEPS) Consortium resulted in identifying key therapeutic development criteria including 1) the development of a regenerative cell therapy that will not only protect cells from ischemic injury but also replace lost and damaged tissues and 2) testing of novel therapeutics in translational animal models that would be more reflective of human pathology with improved predictability^6, 7^. Recent breakthroughs enabling the robust generation of neural stem cells from autologous sources and the development of a robust pig stroke model make it possible to address both of these major criteria^8–10^.

Recent pioneering research has shown that induced pluripotent stem cells (iPSCs) can be generated from a patient’s own somatic cells. These iPSCs have similar plasticity to embryonic stem cells and are able to differentiate into neural stem cells (iNSC) which are used to effectively treat ischemic stroke in rodent models^11, 12^. These studies have demonstrated that upon transplantation, iNSCs have a dual mode of therapeutic action: 1) serving as a cell replacement therapy by differentiating into neurons and glia that integrate into surrounding tissues and 2) producing neuroprotective and regenerative growth factors that stabilize and promote healing of damaged parenchyma^13–16^. In rodent stroke models, transplanted iNSCs reduce microglia activation, glial scar formation, promote angiogenesis, reduce tissue atrophy, and differentiate to mature neuronal cell types that engraft into the host brain^13–15, 17^. However, iNSCs were tested in a rodent brain which, when compared to the human, possesses many cerebral anatomical and physiological differences; therefore, these results may not be directly translatable to clinical applications. Compared to the rodent, the pig brain has greater anatomical and physiological similarities to humans with respect to gray to white matter composition, blood flow, gyral patterning, metabolism, and size - key factors that directly affect injury evolution, tissue recovery and treatment development^18–25^. Therefore, the use of the pig model to develop stroke therapies, particularly iNSC based therapy, could bridge the gap between preclinical rodent models and human clinical trials.

In this study, we report for the first time that intraparenchymal transplantation of human iNSCs improves tissue recovery in the stroke-damaged brain in a pig model. Non-invasive longitudinal magnetic resonance imaging (MRI) of stroked animals provided evidence of recovery in brain metabolism, white matter integrity, and cerebral blood perfusion after iNSC therapy. Histologic assessment showed that iNSC therapy also resulted in neural protection, decreased microglial activation, and stimulated endogenous neurogenesis. Transplanted iNSCs survived for 12 weeks in the pig brain and differentiated into neuronal and glial cells. These results demonstrate that iNSCs have significant potential as a regenerative and cell replacement therapy in a robust translational large animal model, providing further insight into the potential of these cells as a therapeutic for patients.

## Results

### iNSCs express Nestin and Sox1 and differentiate to neurons, astrocytes, and oligodendrocytes *in vitro*

**Figure 1:**
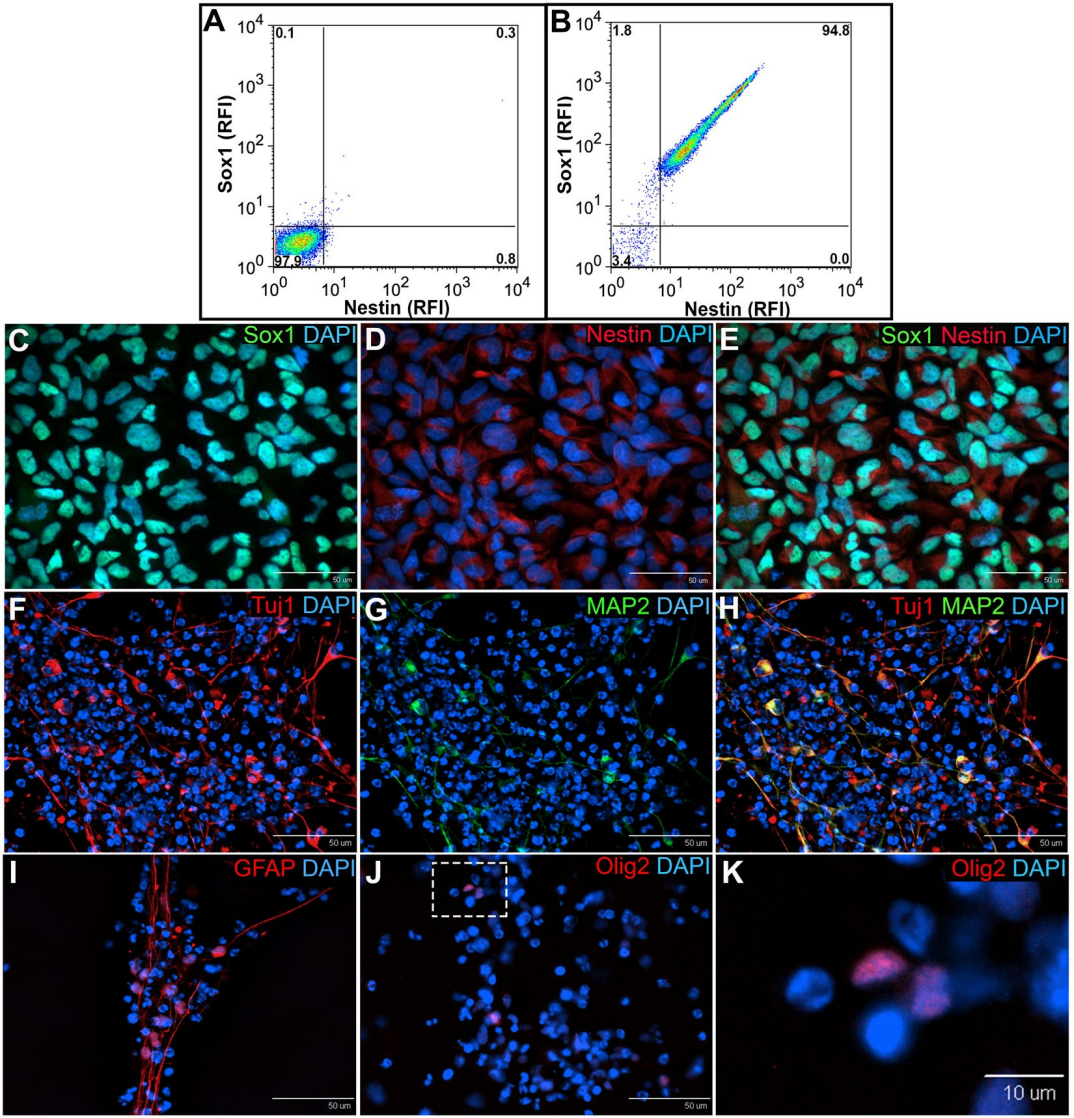
iNSCs express Nestin and Sox1 and differentiate to neurons, astrocytes, and oligodendrocytes *in vitro*. Flow cytometry demonstrated that 94.8% of iNSCs co-expressed the NSC markers Sox1 and Nestin (**B**), while the secondary antibody control was negative for these markers (**A**). Immunocytochemistry of iNSCs confirmed the co-expression of Sox1 (**C**) and Nestin (**D**); co-localization of Sox1 and Nestin (**E**)). iNSCs underwent spontaneous differentiation *in vitro* and were stained for mature neural markers 2 weeks and 4 weeks after bFGF withdrawal. Differentiated iNSCs robustly expressed the neuron markers Tuj1 (**F**) and MAP2 (**G**; co-localization of Tuj1 and MAP2 (**H**)), the astrocyte marker GFAP (**I**) and the oligodendrocyte marker Olig2 (**J,K**). Panel K is the inset represented by the white dotted square in Panel J. RFI = relative fluorescence intensity.

To assess the homogeneity of iNSCs pre-transplantation, flow cytometry and immunocytochemistry were performed for the iNSC markers Sox1 and Nestin. Flow cytometry demonstrated that 94.8% of iNSCs were Sox1+/Nestin+ and immunocytochemistry confirmed consistent homogeneous expression (Fig. 1A–E). Spontaneous differentiation of iNSCs *in vitro* resulted in robust generation of Tuj1+/MAP2+ neurons (Fig. 1F–H) as well as GFAP+ astrocytes and Olig2+ oligodendrocytes (Fig. 1I–K). Together, this data indicates that cultured iNSCs are a true multipotent stem cell population and exhibit robust differentiation into the three major neural lineages: neurons, astrocytes, and oligodendrocytes.

### Stroke results in cerebral swelling followed by atrophy

**Figure 2:**
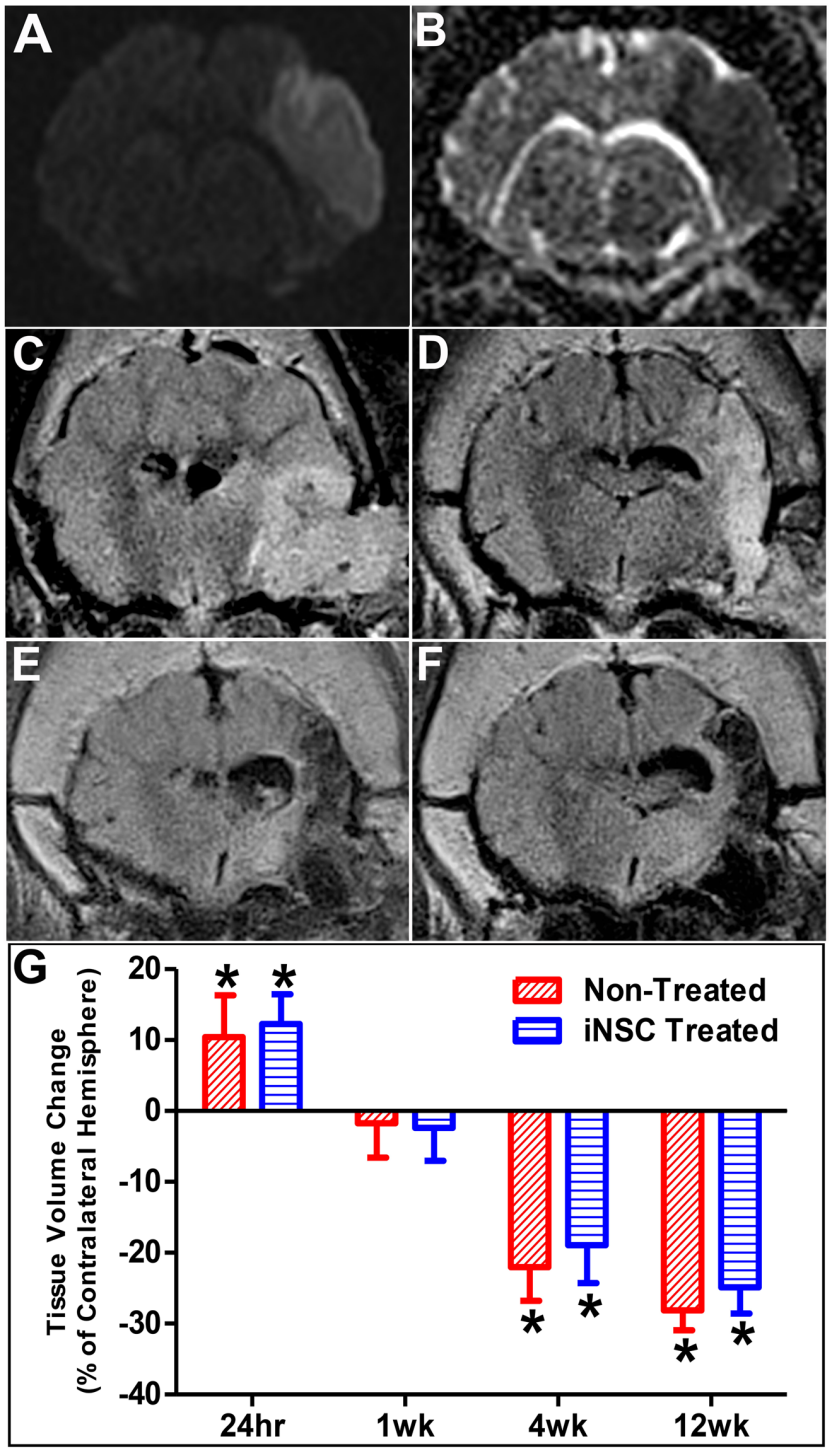
Ischemic Stroke Results in Significant Brain Swelling and Atrophy. MRI was performed 24 hours post-stroke and 1, 4, and 12 weeks post-transplant. Diffusion weighted imaging (DWI) demonstrated abnormal territorial hyperintensity in the right cerebral hemisphere 24 hours post-stroke (**A**). Corresponding ADC maps showed hypointense signal at the lesion site, validating the presence of ischemic stroke (**B**). T2-weighed FLAIR images displayed initial brain swelling in the ipsilateral hemisphere at 24 hours post-stroke (**C**) that subsides by 1 week post-transplant (**D**). T2-weighted FLAIR images taken at 4 weeks (**E**) and 12 weeks (**F**) post-transplant showed significant brain atrophy at the area of the infarct. Volumetric measurements on T1-weighted images supported these changes (**G**). Data are expressed as mean ± s.d. Data is generated from scans of 4 animals per treatment group. *Indicates significance from the volume of the contralateral hemisphere (p < 0.005).

MRI performed 24 hours post-stroke confirmed the presence of cytotoxic ischemia and cerebral infarction. DWI showed abnormal territorial hyperintensity in the right cerebral hemisphere in all 8 pigs indicating the presence of edema (Fig. 2A). ADC maps demonstrated distinct hypointensity at the same region indicative of cytotoxic edema and confirming ischemic stroke (Fig. 2B). A hyperintense territory on T2-weighted FLAIR images 24 hours post-stroke suggested edema formation and resulted in brain swelling in the ipsilateral (injured) hemisphere that shifted the midline towards the contralateral (uninjured) hemisphere, causing the parenchyma and ventricles to be compressed and distorted (Fig. 2C). The ipsilateral hemisphere was significantly (p < 0.005) larger than the contralateral hemisphere in both iNSC treated and non-treated animals (12.30% ± 2.09% and 10.44% ± 2.94%, respectively, Fig. 2G). Five days post-stroke, 5 million iNSCs were transplanted in two injections (for a total of 10 million cells) into the peri-lesional region. The hyperintense lesion persisted 1 week post-transplant with observations of midline shift towards the ipsilateral hemisphere and an enlarged right ventricle due to parenchymal tissue atrophy (Fig. 2D). There was no statistical difference between the ipsilateral and contralateral hemispheres in iNSC treated animals or non-treated animals (−2.39% ± 2.32% and −1.74% ± 2.42%, respectively, Fig. 2G). The lesion site became hypointense on T2-FLAIR by 4 weeks post-transplant due to parenchymal tissue atrophy, and ipsilateral hemisphere was significantly (p < 0.0001) smaller than the contralateral hemisphere in both the iNSC treated and non-treated animals (−18.91% ± 2.70% vs. −22.04% ± 2.37%, respectively, Fig. 2E,G). The hypointense signal persisted through 12 weeks post-transplant, and the ipsilateral hemispheres of both iNSC treated and non-treated groups displayed significant (p < 0.0001) atrophy with a 24.86% ± 1.85% and 28.14% ± 1.40% decrease in volume, respectively (Fig. 2F,G). Although a smaller degree of atrophy was observed in iNSC treated animals, there was no statistical difference between the two treatment groups (Fig. 2G). Taken together, this data indicates that iNSC treatment does not have an effect on parenchymal atrophy after stroke.

### iNSC treatment results in recovered white matter integrity in the chronic stages of stroke recovery

**Figure 3:**
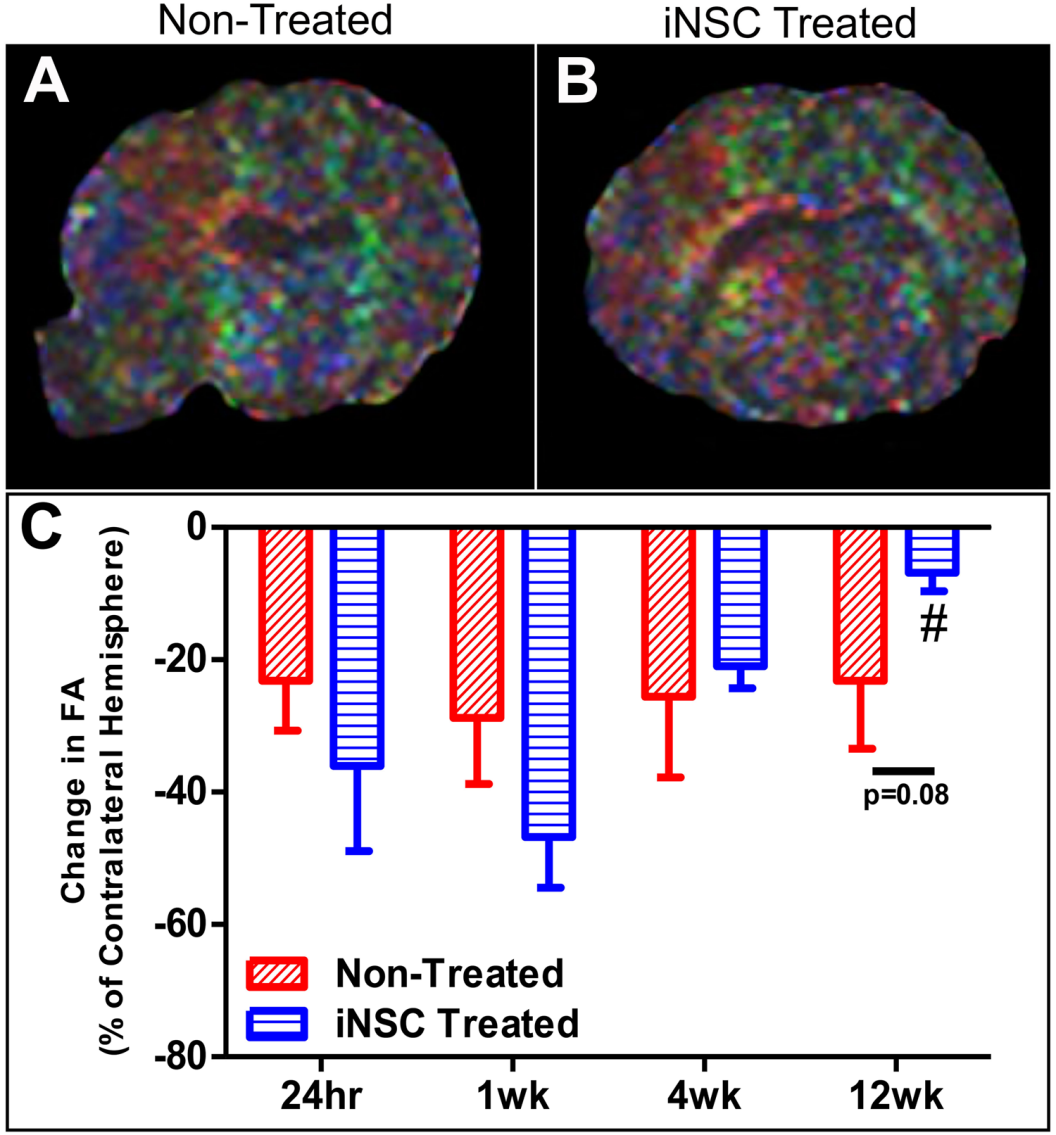
iNSC Treatment Results in Improved White Matter Integrity After Stroke. DTI was performed 24 hours post-stroke and 1, 4, and 12 weeks post-transplant, and FA was calculated within the peri-infarct of the ipsilateral hemisphere and a comparable anatomical region on the contralateral hemisphere. Representative FA maps of non-treated (**A**) and iNSC treated (**B**) animals. The average change in FA in the abnormal territory relative to the contralateral normal control hemisphere was calculated at each time point, and iNSC treatment resulted in enhanced recovery of FA in the ipsilateral hemisphere 12 weeks post-transplant compared to 24 hours post-stroke (**C**). Data are expressed as mean ± s.d. Data is generated from scans of 4 animals per treatment group. ^#^Indicates significance in FA from 24 hours post-stroke within treatment (p < 0.005).

DTI was used to determine whether white matter integrity in damaged tissues could be returned to non-injured, contralateral hemisphere levels after iNSC treatment. Percent changes in fractional anisotropy (FA), a measure of white matter integrity, in the ipsilateral hemisphere were compared to the contralateral hemisphere with calculated changes closer to 0 being more similar to normal tissue (Fig. 3). FA values were increased in selected regions in iNSC-treated animals 12 weeks after the iNSC transplant (Fig. 3A,B). There was a significant (p < 0.005) increase in FA in iNSC treated pigs at 12 weeks post-transplant compared to 24 hours post-stroke (−6.82% ± 2.83% vs. −36.02% ± 12.90%, respectively), while there was no statistical difference between the two time points in non-treated animals (−23.19% ± 10.24% vs. −23.17% ± 7.56%, respectively, Fig. 3C). Furthermore, there was a trending (p = 0.08) decrease in the change in FA in iNSC treated animals compared to non-treated animals at 12 weeks post-injection (−6.82% ± 2.83% and −23.19% ± 10.24%, respectively). This indicates that iNSC treatment leads to more similar FA, and thus white matter integrity, to the normal contralateral hemisphere by 12 weeks post-transplant in the ipsilateral hemisphere.

### iNSC treatment significantly improves recovery of brain metabolism post-stroke

**Figure 4:**
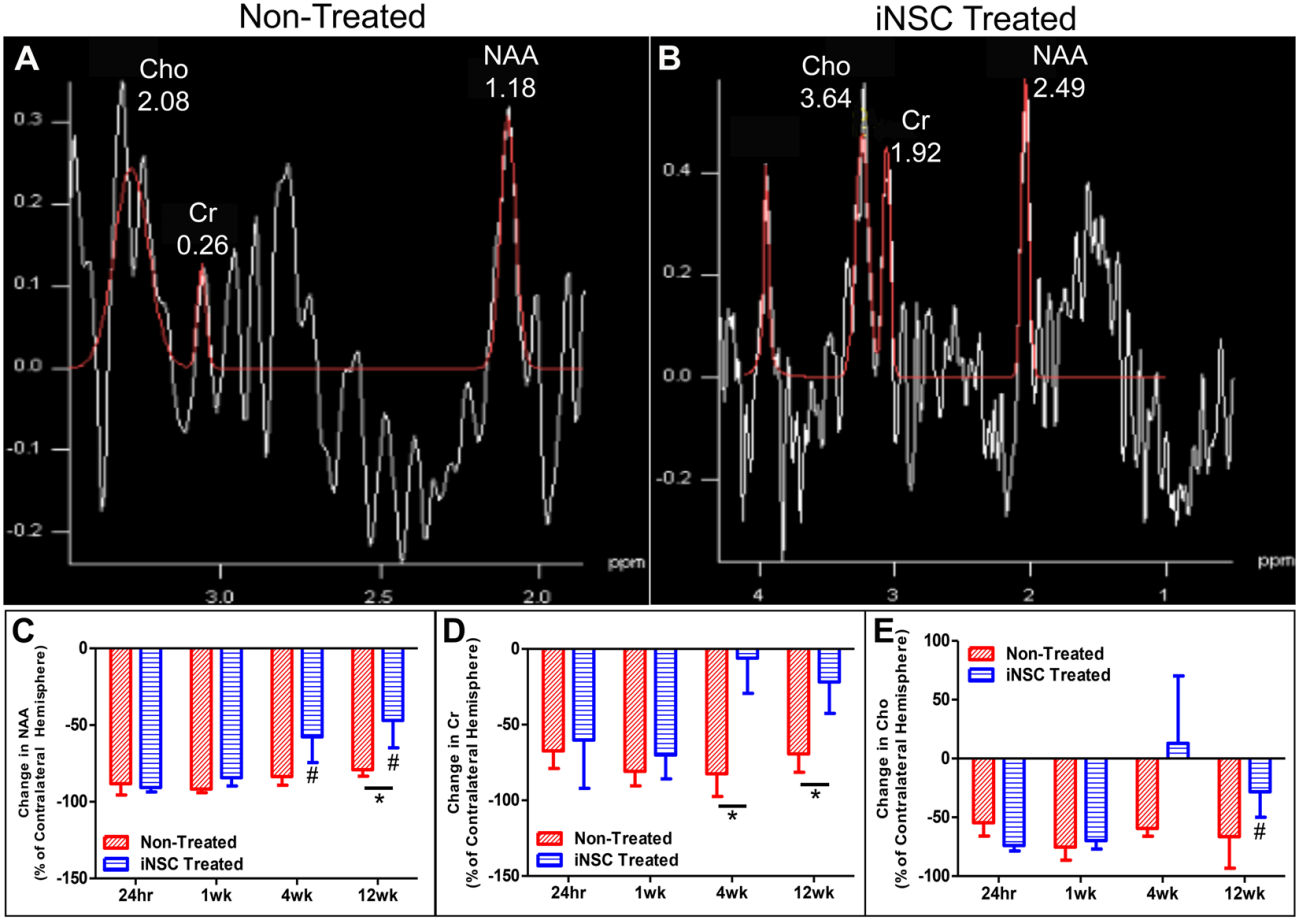
iNSC Treatment Leads to Recovery in Neurometabolite Abundance After Stroke. MRS measured the abundance of the neurometabolites NAA, Cr, and Cho 24 hours post-stroke and 1, 4, and 12 weeks post-transplant. Representative MRS spectra of non-treated (**A**) and iNSC treated (**B**) animals. The average difference in neurometabolite abundance between the ipsilateral and contralateral hemispheres was calculated and expressed as a percentage change in the ipsilateral hemisphere relative to the contralateral hemisphere. iNSC treatment significantly enhanced NAA abundance in the ipsilateral hemisphere 4 weeks and 12 weeks post-transplant compared to 24 hours post-stroke, and NAA abundance was closer to that of the contralateral hemisphere in the iNSC treated animals compared to non-treated animals at 12 weeks post-transplant (**C**). Cr abundance was significantly enhanced in iNSC treated animals compared to non-treated animals at 4 and 12 weeks post-transplant (**D**). Cho abundance was significantly improved in the ipsilateral hemisphere at 12 weeks post-transplant compared to 24 hours post-stroke (**E**). Data are expressed as mean ± s.d. Data is generated from scans of 4 animals per treatment group. *Indicates significance between treatment groups within time point (p < 0.05). ^#^Indicates significance from 24 hours post-stroke within treatment (p < 0.05).

The levels of neurochemicals N-acetylaspartate (NAA, neuronal marker), creatine (Cr, a cell energy metabolite), and choline (Cho, marker of cellularity) are indicative of brain metabolism and can be longitudinally measured by MRS to assess tissue damage and the effect of iNSC treatment (Fig. 4). MR spectra obtained from iNSC treated and non-treated groups showed distinct changes in the levels of NAA, Cr, and Cho (Fig. 4A,B). Peak integrals for each metabolite were measured in both hemispheres and the percent difference in the ipsilateral hemisphere relative to the contralateral hemisphere was calculated to determine the injury and recovery induced changes with changes closer to 0 being more similar to the normal tissue. iNSC treated animals showed significantly (p < 0.01) decreased change in NAA levels relative to non-treated animals at 12 weeks post-transplant of iNSCs (−46.91% ± 17.90% vs. −79.03% ± 4.33%, respectively). Additionally, the change in NAA was significantly (p < 0.0001) decreased at both 4 weeks and 12 weeks post-transplant in iNSC treated animals compared to 24 hours post-stroke (−57.68% ± 16.73% and −46.91% ± 17.90% vs. −90.63% ± 2.93%, respectively), which was not the case for non-treated animals (Fig. 4C). The cell energy metabolite marker Cr demonstrated a similar neurometabolic change with a significant (p < 0.05) reduction in the change in Cr in iNSC treated animals compared to non-treated animals at 4 weeks post-transplant (−6.09% ± 23.25% vs. −82.44% ± 15.02%, respectively) and 12 weeks post-transplant (−21.78% ± 20.83% vs. −69.28% ± 12.12%, respectively) (Fig. 4D). Cho levels showed a significant (p < 0.05) reduction in the change in Cho at 12 weeks post-transplant in iNSC treated animals compared to 24 hours post-stroke (−28.33% ± 21.63% vs. −73.90% ± 4.75%, respectively), which was not observed in non-treated pigs (Fig. 4E). Taken together, this data suggests recovery in brain metabolism with the ipsilateral hemisphere being more similar to the contralateral hemisphere in iNSC treated animals.

### iNSC treatment significantly enhances cerebral blood perfusion in the ipsilateral hemisphere

**Figure 5:**
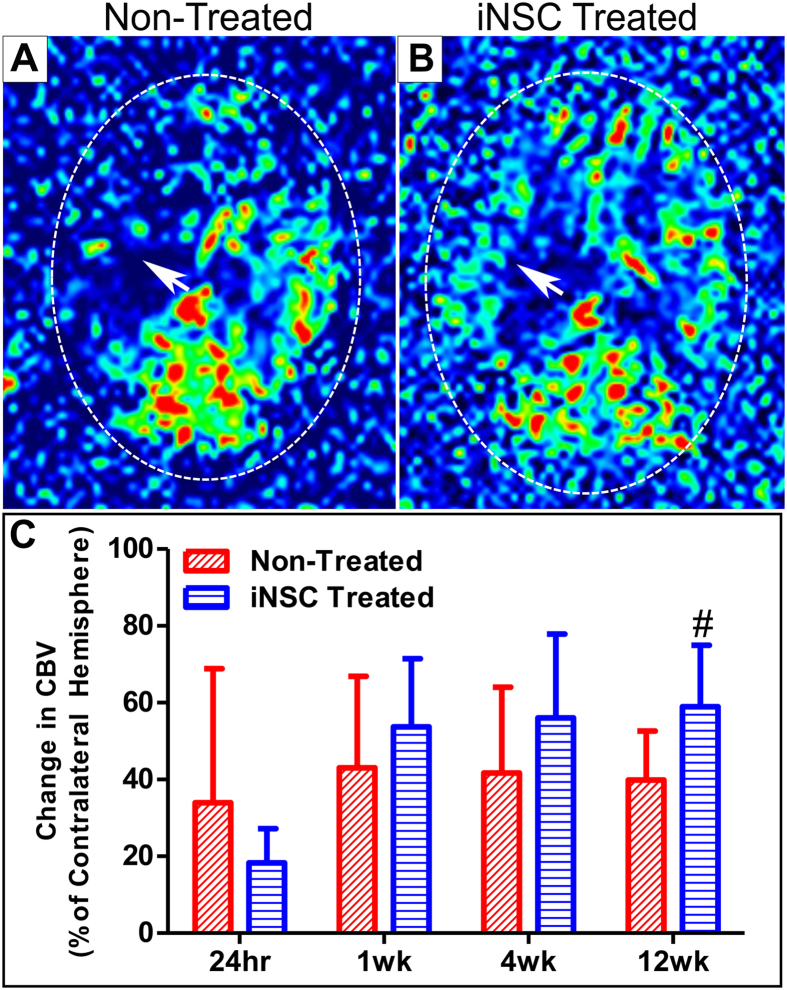
iNSC Treatment Improves Cerebral Blood Perfusion in the Infarcted Area. PWI was utilized to measure CBV in the infarct region. Representative perfusion maps of non-treated (**A**) and iNSC treated (**B**) animals indicating increased cerebral perfusion in the infarct (white arrows) in iNSC treated animals at 12 weeks. CBV was significantly increased in iNSC treated animals 12 weeks post-transplant compared to 24 hours post-stroke (**C**). Data are expressed as mean ± s.d. Data is generated from scans of 4 animals per treatment group. ^#^Indicates significance from 24 hours post-stroke within treatment (p < 0.05).

Perfusion weighted imaging (PWI) was performed to determine if iNSCs could normalize blood perfusion to the brain after stroke. Color-coded cerebral perfusion maps taken at 12 weeks post-transplant suggest increased cerebral blood flow at the area of the infarct in iNSC treated pigs compared to non-treated pigs (Fig. 5A,B). Cerebral blood volume (CBV) was calculated in both hemispheres, and the measurement of the affected ipsilateral hemisphere was expressed as a percentage of the normal contralateral hemisphere for each parameter. CBV quantifies the volume of blood within a particular volume of brain tissue; a decrease in CBV indicates decreased perfusion within a volume of brain tissue^26, 27^. CBV was found to be significantly (p < 0.05) increased in iNSC treated animals at 12 weeks post-transplant compared to 24 hours post-stroke (58.96% ± 15.98% vs. 18.31% ± 8.87%, respectively), which was not observed in non-treated animals (Fig. 5E). This data indicates that iNSC treatment enhances cerebral blood perfusion in the ipsilateral hemisphere by 12 weeks post-transplant.

### iNSC treatment leads to neuronal protection and reduced microglial activation at the lesion border

**Figure 6:**
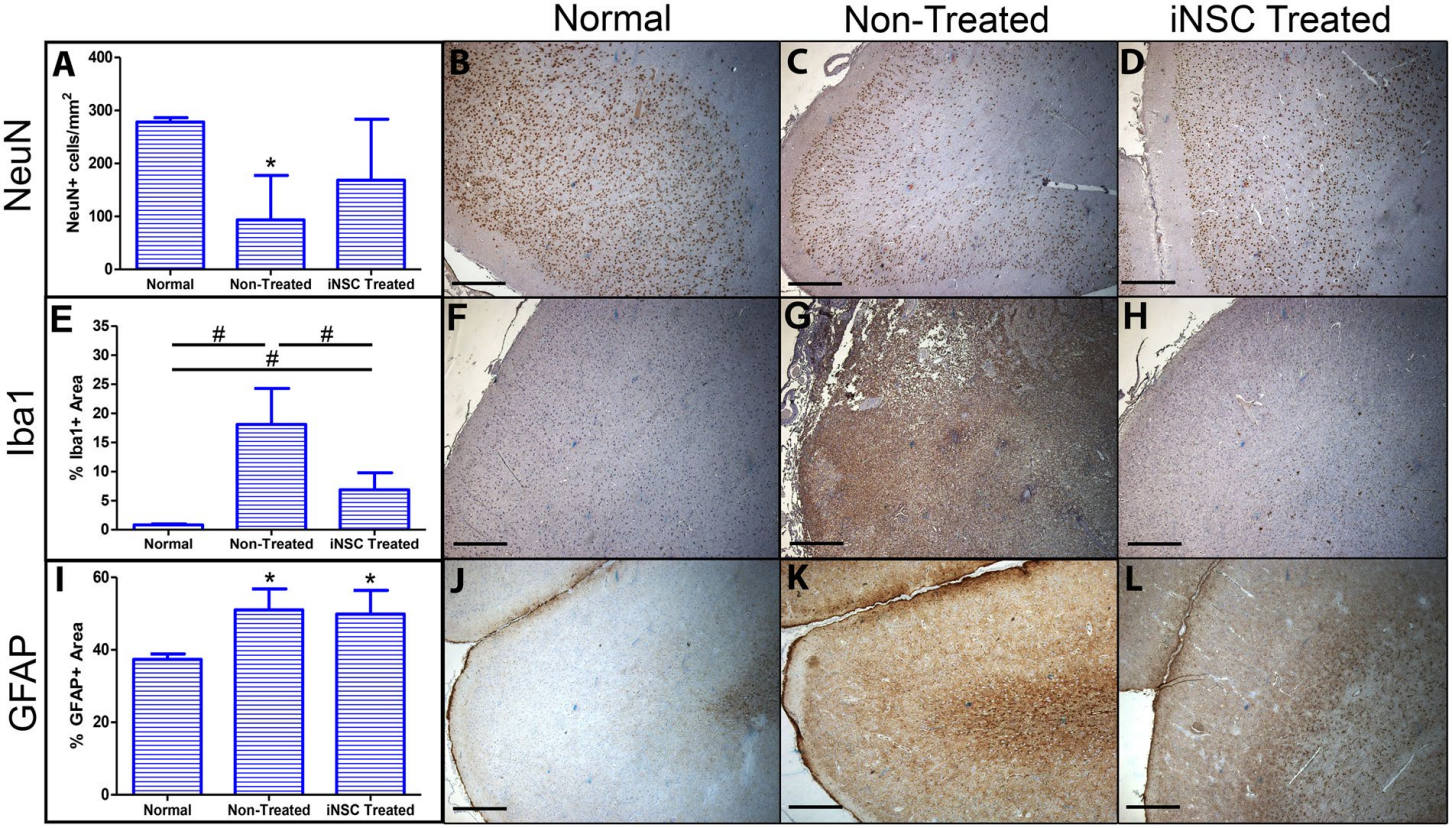
iNSC Treatment Aids in the Protection of Neurons and Reduces Microglia Activation at the Lesion Border. Quantification of NeuN+ cells/mm^2^ at the lesion border shows significant reduction of neurons in non-treated animals but no significant change between normal controls and iNSC treated animals (**A**). Representative image of NeuN+ neurons at the lesion border in normal control (**B**), non-treated (**C**), and iNSC treated (**D**) animals. Quantification of the percentage Iba1+ area at the lesion border shows a significant increase in Iba1+ area in stroked animals compared to normal control animals and a significant reduction in Iba1+ area in iNSC treated animals compared to non-treated animals (**E**). Representative image of Iba1+ microglia at the lesion border in normal control (**F**), non-treated (**G**), and iNSC treated (**H**) animals. Quantification of the percentage GFAP+ area demonstrates a significant increase in GFAP+ area in both non-treated and iNSC treated groups compared to normal control animals (**I**). Representative image of GFAP+ astrocytes at the lesion border in normal control (**J**), non-treated (**K**), and iNSC treated (**L**) animals. Data are expressed as mean ± s.d. *Indicates significance from normal controls (p < 0.05). Data is generated from one brain section from each animal (n = 4 per treatment group). ^#^Indicates significance between treatment groups (p < 0.05). Scale bars, B-D, F-H, J-L, 500 μm.

Next, we sought to determine the effect of iNSC transplantation on neuronal survival and immune response after stroke. At 12 weeks post-transplant, there was a significant (p < 0.01) decline in the number of NeuN+ neurons in non-treated animals at the lesion border compared to normal control animals (93 ± 84 cells/mm^2^ vs. 278 ± 9 cells/mm^2^, respectively). However, there was no significant difference in the number of NeuN+ neurons between iNSC treated and normal control animals (169 ± 115 cells/mm^2^ vs. 278 ± 9 cells/mm^2^, respectively) at the lesion border, indicating that iNSC treatment leads to neuronal protection at the lesion site post-stroke (Fig. 6A–D).

In addition, we assessed immune response by quantifying microglia activation and astrogliosis at the lesion border through Iba1 and GFAP antibody staining, respectively (Fig. 6E–L). 12 weeks post-transplant, there was a significant (p < 0.01) increase in Iba1+ area in both iNSC treated and non-treated animals compared to normal control animals (6.88% ± 2.91% and 18.10% ± 6.17% vs. 0.85% ± 0.16%, respectively). Remarkably, there was a significant (p < 0.05) reduction in Iba1+ area in iNSC treated animals compared to non-treated animals (6.88% ± 2.91% vs. 18.10% ± 6.17%, respectively) demonstrating that iNSC treatment mitigates microglia activation after stroke at the lesion border (Fig. 6E–H). Astroglial activity at the lesion border was significantly (p < 0.05) upregulated as indicated by the GFAP+ area in both iNSC and non-treated pigs compared to normal control animals (49.89% ± 6.55% and 51.04% ± 5.79% vs. 37.39% ± 1.47%, respectively). However, there was no significant difference in GFAP+ area between iNSC treated and non-treated treated groups suggesting that iNSC treatment does not have an effect on astrogliosis after stroke at the lesion border (Fig. 6I–L). Taken together, this data indicates that iNSC treatment likely has a neuroprotective effect leading to decreased neuronal cell death in cortical regions of the brain and suppressed microglia activation.

### iNSC treatment promotes endogenous neuroblast activation and migration to the lesion border

**Figure 7:**
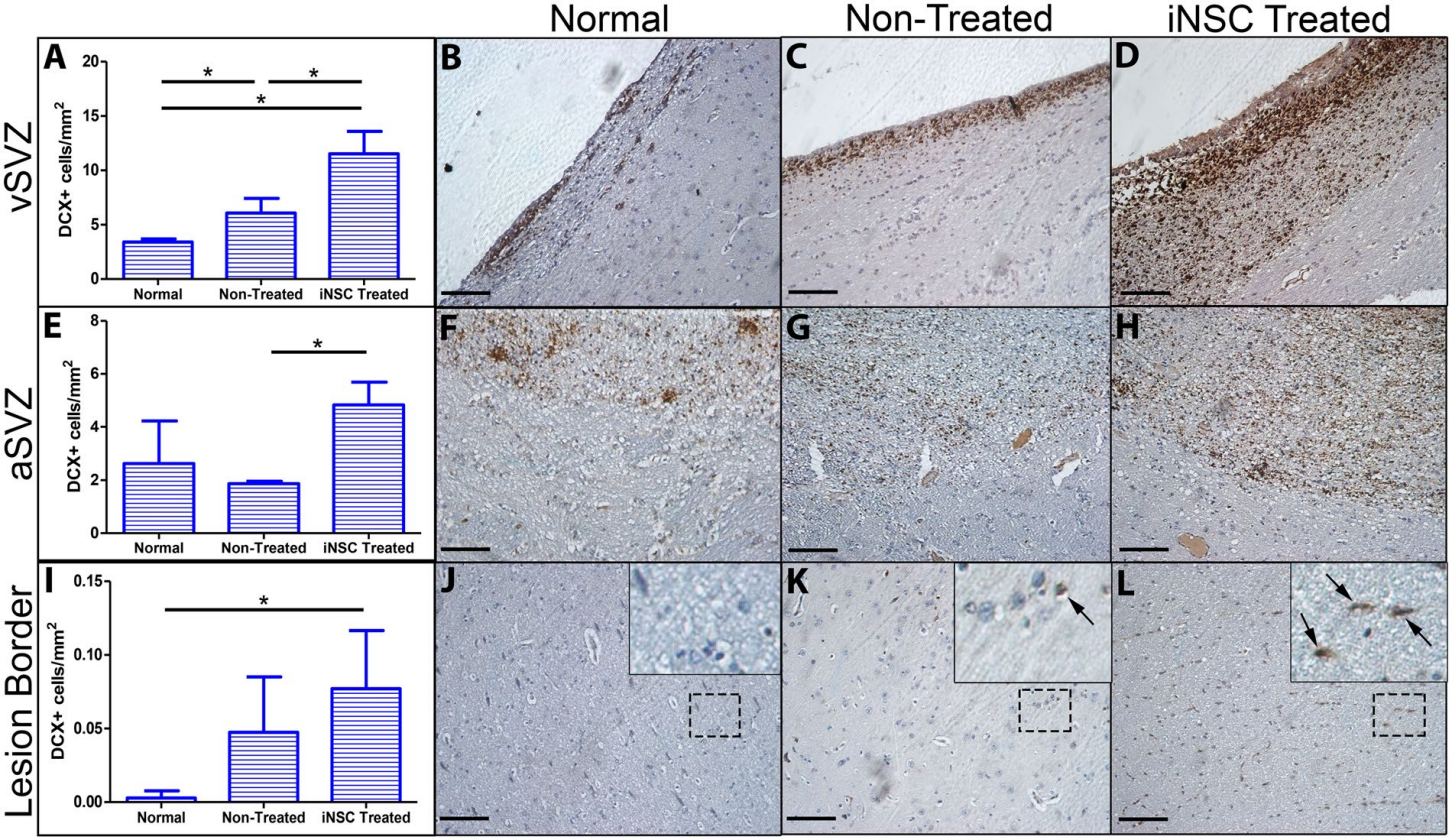
iNSC Treatment Promotes Endogenous Neuroblast Proliferation and Migration to the Lesion Border. Quantification of DCX+ cells/mm^2^ in the vSVZ demonstrated a significant increase in DCX+ neuroblasts in non-treated animals compared to normal control animals and a significant increase in DCX+ neuroblasts in iNSC treated animals compared to non-treated animals (**A**). Representative image of DCX+ neuroblasts at the vSVZ in normal control (**B**), non-treated (**C**), and iNSC treated (**D**) animals. Quantification of DCX+ cells/mm^2^ in the abventricular subventricular zone (aSVZ) showed a significant increase in DCX+ neuroblasts in iNSC treated animals compared to non-treated animals (**E**). Representative images of DCX+ neuroblasts at the aSVZ in normal control (**F**), non-treated (**G**), and iNSC treated (**H**) animals. Quantification of DCX+ cells/mm^2^ at the lesion border demonstrated a significant increase in DCX+ neuroblasts in iNSC treated animals compared to normal control animals (**I**). Representative image depicting minimal DCX+ neuroblasts in normal control animals (**J**). Representative image of DCX+ neuroblasts (black arrows) in non-treated (**K**) and iNSC treated (**L**) animals. Black dotted square represents inset in J-K. Data are expressed as mean ± s.d. Data is generated from one brain section from each animal (n = 4 per treatment group). *Indicates significance between treatment groups (p < 0.05). Scale bars, B-D, F-H, J-L, 100 μm.

After stroke, endogenous neural stem cells located in the subventricular zone (SVZ) have the ability to differentiate into immature neuronal cells, or neuroblasts, which migrate toward the site of injury replacing damaged and lost cells (albeit at a low level) and have a neuroprotective effect through the release of neurotrophic factors^28^. Specifically in the pig, Costine *et al*. has characterized a process in which neuroblasts in the SVZ proliferate at the ventricular lining of the SVZ (vSVZ) before forming migratory chains directly adjacent to the vSVZ, deemed the abventricular SVZ (aSVZ)^29^. From the aSVZ, neuroblasts migrate toward the site of neural injury. We wanted to determine if neuroblasts in our pig MCAO model demonstrated this same migratory pattern, and if iNSC treatment promoted neuroblast migratory behavior from the SVZ. 12 weeks post-transplant, there was a significant (p < 0.05) increase in DCX+ neuroblasts at the level of the vSVZ in non-treated animals compared to normal control animals (6.08 ± 1.35 cells/mm^2^ vs. 3.40 ± 0.29 cells/mm^2^, respectively). Furthermore, there was a significant (p < 0.01) increase of DCX+ neuroblasts in the vSVZ in iNSC treated animals compared to non-treated animals (11.52 ± 2.07 cells/mm^2^ vs. 6.08 ± 1.35 cells/mm^2^, respectively) (Fig. 7A–D). When assessing the amount of DCX+ neuroblasts in the aSVZ, we found no significant difference between non-treated and normal control animals (1.87 ± 0.10 cells/mm^2^ vs. 2.63 ± 1.59 cells/mm^2^, respectively). However, there was a significant (p < 0.01) increase in DCX+ neuroblasts in the aSVZ in iNSC treated animals compared to non-treated animals (4.83 ± 0.86 cells/mm^2^ vs. 1.87 ± 0.10 cells/mm^2^, respectively) (Fig. 7E–H). Last, we compared the amount of DCX+ neuroblasts between treatment groups at the lesion border and found no significant difference in the number of DCX+ neuroblasts between non-treated animals and normal control animals (0.047 ± 0.038 cells/mm^2^ vs. 0.003 ± 0.005 cells/mm^2^, respectively). However, we found significant (p < 0.05) increase in the amount of DCX+ neuroblasts at the lesion border in iNSC treated animals compared to normal control animals (0.077 ± 0.040 cells/mm^2^ vs. 0.003 ± 0.005 cells/mm^2^, respectively) (Fig. 7I–L). These results indicate that stroke alone stimulates neurogenesis at the vSVZ; however, iNSC treatment significantly augments this neurogenic activity and promotes neuroblast migration to the aSVZ and the lesion border.

### Transplanted iNSCs survive long-term and differentiate into neurons and oligodendrocytes

**Figure 8:**
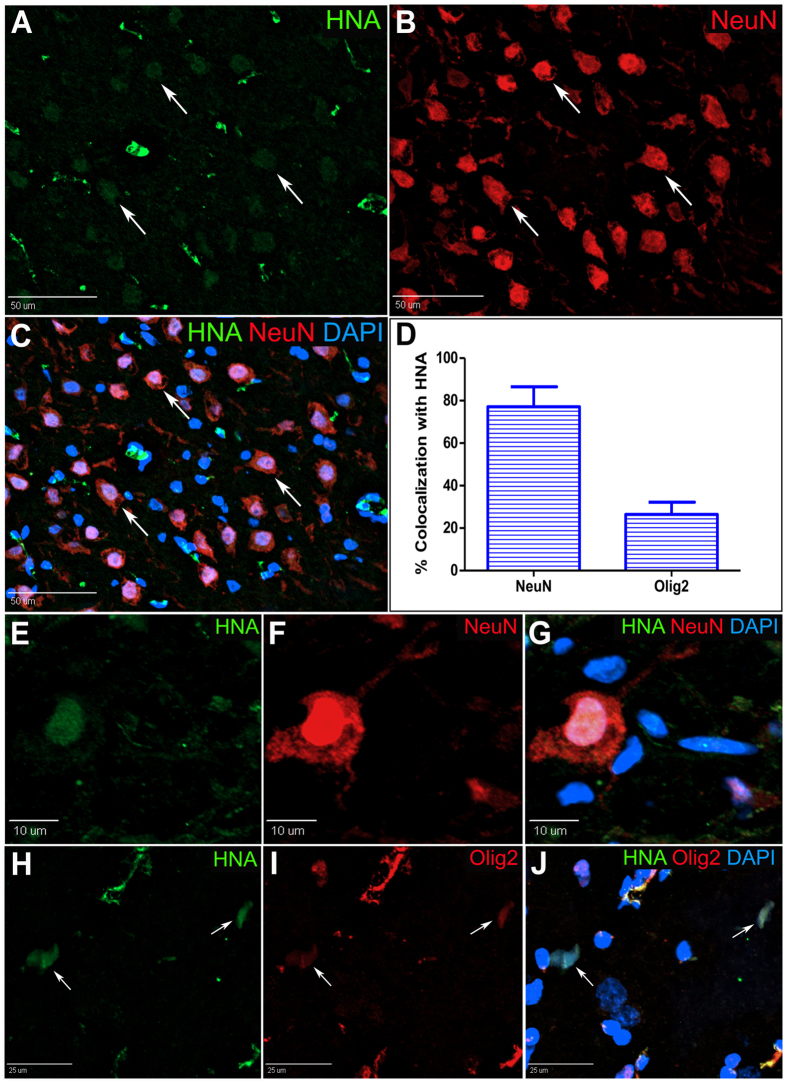
iNSCs Survive Long-Term and Differentiate into Neurons and Oligodendrocytes in the Pig Brain After Stroke. Pigs were sacrificed at 12 weeks post-transplant, and transplant sites underwent immunohistochemistry to characterize iNSC differentiation. Representative image of HNA+ human cells in the brain parenchyma co-localizing with the mature neuron marker NeuN (**A–C**, white arrows). Quantification of HNA+ cells co-localized with either NeuN or Olig2 revealed both neurons and oligodendrocyte populations derived from transplanted iNSCs (**D**). Representative image of HNA+ cell colocalized with NeuN displaying distinct neuronal morphology (**E**–**G**). Representative images of HNA+ cells colocalized with the oligodendrocyte marker Olig2 (**H**–**J**). Data are expressed as mean ± s.e.m. Data is generated from one brain sample from each iNSC treated animal (n = 4).

Transplanted human iNSC differentiation in the post-stroke pig brain was assessed by immunohistochemistry (Fig. 8). Transplanted human cells were identified by expression of the human nuclear antigen (HNA, Fig. 8A). HNA+ cells were co-labeled with either NeuN, Olig2, or GFAP to identify transplanted cells that differentiated to neurons, oligodendrocytes, or astrocytes, respectively. Co-labeling HNA+/NeuN+ cells made up the majority of the HNA+ cells (Fig. 8A–G). A subset of HNA+ cells also co-localized with Olig2 (Fig. 8H–J). The percentage of HNA+ cells that co-localized with either NeuN or Olig2 was 77.15% ± 18.8% and 26.45% ± 11.54%, respectively (Fig. 8D). Due to the nature of the GFAP label, it was difficult to confidently identify HNA+/GFAP+ cells; therefore, we did not include HNA+/GFAP+ cells in our quantification. This data shows that transplanted iNSCs differentiate predominantly into neurons. Furthermore, we show evidence of substantial iNSC differentiation into oligodendrocytes, likely the result of utilizing an animal model with a high percentage of white matter.

### iNSC treatment leads to distinct changes in gene expression related to tissue recovery in gray and white matter

**Figure 9:**
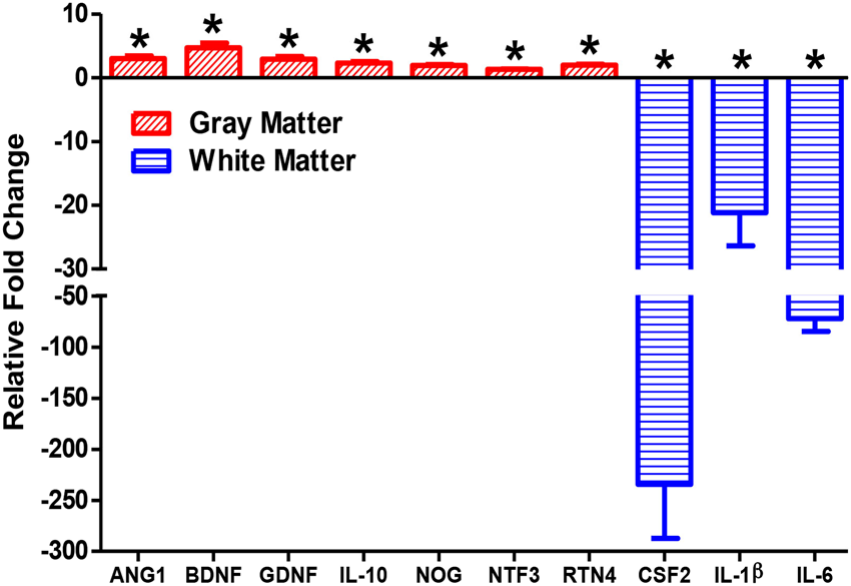
iNSC Treatment Leads to Distinct Changes in Gene Expression Related to Tissue Recovery in Gray and White Matter. Quantitative RT-PCR was performed on iNSC treated and non-treated gray and white matter tissue samples. Graph represents relative fold change of iNSC treated gray and white matter relative to non-treated gray and white matter samples. There was a significant upregulation in the expression of the genes ANG1, BDNF, GDNF, IL-10, NOG, NTF3, and RTN4 in iNSC treated gray matter samples relative to non-treated gray matter samples. There was a significant downregulation in the expression of the genes CSF2, IL-1β, and IL-6 in iNSC treated white matter samples relative to non-treated white matter samples. Data are expressed as mean ± s.e.m. *Indicates significance from non-treated sample within tissue type (p < 0.05). Data is generated from one brain sample from each animal (n = 4 per treatment group).

Many studies have shown that transplanted iPS- and fetal-derived NSCs produce regenerative trophic factors that decrease inflammation, promote angiogenesis, and aid in tissue regeneration after stroke^13, 14, 17, 30, 31^. We performed quantitative RT-PCR to determine if iNSC treatment resulted in changes in gene expression related to tissue recovery (Fig. 9). We analyzed gray and white brain matter separately to determine whether the two tissue types responded differently to signaling from transplanted iNSCs. After 12 weeks post-transplant, several Sus scrofa-specific genes related to angiogenesis, neurotrophism, and inflammation were significantly (p < 0.05) changed in iNSC treated brain tissue relative to non-treated brain tissue. Furthermore, gray and white matter exhibited distinct changes in gene expression compared to one another. In gray matter, the angiogenesis marker Ang1, neurotrophic factors BDNF and GDNF, anti-inflammatory cytokine IL-10, growth factors NOG and NTF3, and neurite branching mediator Rtn4 were significantly (p < 0.05) upregulated in iNSC treated animals by 3.01 ± 0.82, 4.68 ± 1.49, 2.92 ± 0.81, 2.31± 0.43, 1.90 ± 0.31, 1.32 ± 0.09, and 1.98 ± 0.30 fold, respectively, relative to non-treated animals. In white matter, the genes encoding colony stimulating factor 2 (CSF2) and inflammatory cytokines Il-1β and IL-6 were significantly downregulated in iNSC treated animals by 234.33 ± 105.85, 21.19 ± 10.38, and 71.74 ± 25.21 fold, respectively, relative to non-treated animals. Taken together, this data indicates that iNSC treatment leads to changes in gene expression that decreases inflammation and promotes tissue recovery. Interestingly, the changes in gene expression were dissimilar between gray and white matter indicating that gray and white matter responds differently to iNSC trophic factor signaling.

## Discussion

The potential to perform autologous or allogeneic iNSC transplantation to replace and protect brain tissue has significant implications for the treatment of stroke, with promising rodent studies demonstrating drastic improvements in recovery. However, the enthusiasm of the preclinical success of iNSC therapy has been tempered with the hundreds of failed clinical trials of therapeutics previously developed in rodents and a need for a deeper understanding of the underlying recovery mechanisms^5^. For the first time, we demonstrate in a pig stroke model utilizing translational approaches (MRI and MRS) that iNSC cell therapy leads to significant tissue recovery and cell replacement. This tissue level recovery strongly correlated with decreased immune (microglia) response and increased neuroblast migration, demonstrating that the iNSC recovery mechanism is both neuroprotective and regenerative. In addition, iNSCs demonstrated long term integration with robust cell differentiation into both neurons and oligodendrocytes. These results demonstrate that iNSCs are a multi-modal therapeutic with neuroprotective, regenerative, and cell replacement capabilities.

Interestingly, we found that iNSC treatment promotes neurogenesis and the migration of DCX+ neuroblasts from the SVZ to the lesion border. Previous reports have shown that in addition to the SVZ proper (vSVZ), pigs possess an extensive aSVZ containing neuroblast chains extending laterally similar to human infants and other mammalian species which migrate toward the lesion after neural injury^29, 32^. Neurogenesis after NSC transplantation has been well documented in rodent stroke models. These studies showed that hESC- and fetal-derived NSC treatment enhances cell proliferation and migration in the SVZ, DG, and striatum, and a subset of these cells co-label with the neuroblast marker DCX and the mature neuron marker Fox3, indicating endogenous neurogenesis mediated by NSC transplantation may contribute to neuronal replacement in the stroke-damaged brain^33–39^. In the current study we provide evidence that iNSCs exhibit similar neurogenic behavior in the stroke-damaged pig brain. To our knowledge, we are the first to provide evidence of how stroke and iNSC therapy affects neurogenic activity in the SVZ in a gyrencephalic species. Stroke alone stimulated neurogenesis at the vSVZ, but iNSC treatment augmented the neurogenic activity at the vSVZ and was important for increased neuroblast migration to the aSVZ and lesion border, likely through enhanced trophic factor signaling mediated by transplanted iNSCs. Indeed, BDNF and NOG, the gene encoding Noggin, expression was upregulated in brain tissue of iNSC treated animals and have both been shown to stimulate neurogenesis from progenitor cells in the SVZ^28, 40, 41^. These results support a strong paracrine-mediated endogenous neurogenesis role of the transplanted iNSCs.

We found a higher number of neurons at the lesion border in iNSC treated animals compared to non-treated animals 12 weeks post-transplantation. Due to the limited number of identified engrafted iNSCs and migratory endogenous neuroblasts, it is unlikely that neuronal replacement would have such a profound effect on neuronal number by this time point. Therefore, we hypothesize that the observed increase in neurons at these sites is probably due to neuronal protection by iNSC trophic factor signaling. Polentes *et al*. demonstrated TH+ neuronal protection in the substantia nigra one month post-iNSC transplantation, an effect attributed to unknown iNSC-derived trophic factors acting on host substantia nigra afferents^14^. In the current study, we found increased expression of the neurotrophic factors BDNF, GDNF, and NTF3 in the brain tissue of iNSC treated animals, which have been shown to protect neurons from mechanisms related to ischemic insult^42–44^. Furthermore, we found increased expression of the gene encoding neurite branching mediator Rtn4 in iNSC treated brain tissue, which further indicates that iNSCs may foster neural repair mechanisms after stroke.

Neuroinflammation is a hallmark of stroke pathology and is a component of the secondary injury cascade in both human and preclinical animal models^45–47^. Microglia, the resident macrophages of the brain, proliferate and migrate to the lesion where they produce cytotoxic substances that exacerbate brain injury after an ischemic event^48^. Clinical studies have shown that the pharmacologic amelioration of microglia activity improves outcome after stroke^49, 50^. Our data indicates that iNSC treatment significantly reduced the number of microglia at the lesion border by 12 weeks post-transplant, thereby diminishing pro-inflammatory signaling. Indeed, we found a significant decrease in the expression of pro-inflammatory cytokines CSF2, IL-1β, and IL-6 in the brain tissue of iNSC treated animals^51, 52^. Similar to our findings, Huang *et al*. and Chang *et al*. documented reduced microglia activity in rodent stroke models after fetal-derived and iPSC-derived NSC treatment, respectively^13, 17, 30^. However, we found no iNSC-mediated effects on astrogliosis, another hallmark of cerebral secondary injury, in our model. Similarly, Oki *et al*. found that iNSC transplantation did not influence the number of activated astrocytes after stroke^13^. Although reduced astrogliosis after iNSC therapy has been documented in other rodent models of stroke, it is possible that the cell line tested in this study, or other therapeutic factors such as cell dose or treatment window, did not possess the required signaling capabilities to mitigate astrogliosis in the pig brain after stroke^17^.

We demonstrate that our transplanted iNSCs differentiate into both neuronal and glial phenotypes by 12 weeks post-transplantation. The majority of the transplanted cells co-localized with the mature neuron marker NeuN while a smaller subset co-localized with the oligodendrocyte maker Olig2. Our data correlates with previous reports in rodents showing that transplanted iNSC preferentially take on a neuronal phenotype^13–15, 17^. Other groups have demonstrated astrocytic differentiation by iNSCs; however, we are the first to show evidence of robust oligodendrocyte differentiation by iNSCs after transplantation in a preclinical large animal stroke model^17, 36, 53–56^. We hypothesize that this finding may be attributed to the environmental signals inherent to a gyrencephalic brain with abundant white matter; external cues specifically mediated in white matter compartments may induce iNSCs to differentiate toward an oligodendrocyte fate^56, 57^. Future studies are needed to elucidate the different signaling mechanisms needed to induce differentiation to region-specific phenotypes after iNSC transplantation *in vivo*.

Cerebral blood perfusion status is believed to be the most important factor in the clinical management of stroke because restoration of regional perfusion provides substantial help in salvaging endangered ischemic brain tissue^3^. In the present study, stroke-induced deficits in CBV were significantly increased by iNSC therapy by 12 weeks post-transplantation. Previous studies have shown that NSC transplantation promotes the expression of the angiogenesis factor VEGF in endogenous tissues and augments angiogenic activity at the lesion border in rodent stroke models, resulting in improved perfusion^13, 35, 37, 38, 58, 59^. In the present study, we observed a significant increase in the expression of the angiogenesis marker Ang1 12 weeks after iNSC treatment, which may in part explain the observed improvement in cerebral perfusion after iNSC treatment. Previous reports show that transplantation of the fetal NSC cell line CTX0E03, which highly expresses the Ang1 protein, increases the amount of microvessels in the stroked rat brain. A recent Phase I clinical trial testing CTX0E03 (PISCES) noted functional improvement after treatment^60^. Thus, the expression of Ang1 coupled with improved cerebral perfusion in our model suggests increased cerebral microvessel density in the peri-lesional area, which could improve functional recovery. Future studies are needed to further assess the mechanism of this improvement in cerebral vascular performance post-stroke.

We also demonstrate that iNSC therapy leads to more similar FA values, an indicator of white matter integrity, and brain metabolism in the ipsilateral hemisphere compared to the contralateral hemisphere by 12 weeks post-transplantation. Disruption of the myelinated and unmyelinated axon bundles present in white matter impairs communication between neurons in gray matter regions, often leading to motor dysfunction, neurobehavioral syndromes, and cognitive impairment^20^. Previous studies in rodent stroke models aimed to recapitulate white matter injury demonstrate that stem cell treatment facilitates white matter reorganization, enhances connectivity, and improves FA through increases in myelination, axonal sprouting, and white matter bundle thickness in the ischemic boundary^61–66^. The use of the rodent model in these studies possesses certain limitations as the rodent brain contains only 14% white matter, while the human brain contains >60%; therefore white matter repair may be achieved more easily in the rodent than the human^20^. Furthermore, the PISCES trial demonstrated that NSC treatment increased FA value in white matter, a similar result observed in this study^60^. MRS results demonstrated recovery in neurometabolites NAA, Cr, and Cho in iNSC treated animals, complementing our histological findings that iNSC treatment enhances neuronal survival and promotes tissue recovery. Previous studies in rodents demonstrate increased NAA abundance in the stroked hemisphere mediated by adult stem cell trophic factor signaling correlated with improved functional outcome^67, 68^. These results suggest notable enhancement in white matter structural and total brain metabolic recovery due to iNSC therapy.

In conclusion, we show for the first time in a translational large animal pig stroke model that iNSC therapy improves recovery through a combination of neuroprotective, regenerative, and cell replacement mechanisms resulting in improved white matter integrity, cerebral perfusion, and brain metabolism at the tissue level. These macro-level tissue changes in recovery were mechanistically supported with cellular level changes with iNSC treatment leading to reduced immune response, enhanced neurogenesis, and increased neuron survival. Finally, iNSCs survive long-term and differentiate predominantly into neurons. With the increased predictive potential of testing iNSC therapy in a gyrencephalic pig stroke model, these findings support that iNSC therapy may serve as a robust therapeutic in human patients and are deserving of further study.

## Methods

### Human Induced Pluripotent Stem Cell-Derived Neural Stem Cells (iNSC)

Human induced pluripotent stem cell-derived neural stem cells (HIP^TM^ hNSC BC1, GlobalStem^®^, Rockville, MD; hereafter “iNSC”) were used for transplantation into the pig brain. Details for iNSC culture, flow cytometry, immunocytochemistry, and *in vitro* differentiation can be found in Supplementary Methods.

### Labeling of iNSCs

Immediately prior to transplantation, low passage (passage <15) iNSCs were labeled with the lipophilic tracer 1,1′-dioctadecyl-3,3,3′,3′-tetramethylindotricarbocyanine iodide (DiR; Invitrogen) for *in vivo* tracking. Details can be found in Supplementary Methods.

### Pig Stroke Model

All experimental protocols performed in this study were reviewed, approved, and complied with the guidelines established by the University of Georgia Institutional Animal Care and Use Committee. Eight commercially bred, male castrated Landrace pigs aged 6 months and weighing approximately 180 lbs were obtained from the University of Georgia Swine Farm two weeks prior to surgery. The pig stroke model was prepared based on the procedure described previously and permanent middle cerebral artery occlusion (MCAO) was performed in all animals^9^. Details can be found in Supplementary Methods.

### Magnetic Resonance Imaging

Magnetic resonance imaging (MRI) was performed 24 hours post-stroke and 1, 4, and 12 weeks post-transplantation on a Siemens 1.5 Tesla MRI system (Magnetom Avanto, Siemens Healthcare System). Under the same anesthesia protocol utilized for surgery, MRI of the cranium was performed using an 8-channel head coil with the animal positioned in dorsal recumbency. Standard multiplanar MR brain imaging series at the coronal plane were acquired, including T1-weighted, T2-weighted, T2-weighted fluid attenuated inversion recovery (FLAIR), diffusion-weighted imaging (DWI), diffusion tensor imaging (DTI), dynamic susceptibility contrast perfusion-weighted imaging (PWI), and magnetic resonance spectroscopy (MRS). For PWI, the contrast agent (Magnevist^®^, 20 mL, IV) was administered at a rate of 2 mL/second. Researchers were blinded to treatment group assignment when performing quantification analysis. Details can be found in Supplementary Methods.

### iNSC Transplantation

Five days after MCAO surgery, all pigs were alternately assigned to either iNSC treated or non-treated (vehicle only) groups and were anesthetized under the same protocol utilized for the surgery. The surgery site was then reopened for iNSC transplantation into the brain parenchyma. Transplantation surgeries were performed utilizing a large animal stereotaxic frame (David Kopf Instruments) with modifications to meet our needs with pigs. The frame is equipped with a mounted Quintessential stereotaxic injector (Stoelting Co., Wood Dale, IL) to regulate cell injection location and parameters. Previous studies in our laboratory have shown that the use of immunosuppressive drugs did not improve iNSC survival in the pig brain (unpublished data); therefore, the use of immunosuppression was omitted in this study. The iNSC transplantation portion of this study was carried out in a single-blinded manner in which the researchers were aware of treatment group assignment. Details can be found in Supplementary Methods.

### Brain Tissue Collection and Processing

Twelve weeks after MCAO surgery, all eight pigs were sedated utilizing the pre-operative drug protocol and immediately euthanized by intravenous pentobarbital overdose (1 mL/4.5 kg). Brains were removed and coronally sectioned using a brain slicer specific to pig (Zivic Instruments, Pittsburgh, PA). Details for tissue processing can be found in Supplementary Methods.

### Immunofluorescence of iNSC Transplantation Sites

Brain tissue samples were cryosectioned (Leica CM1950) at a thickness of 10 μm and collected on gelatin-coated microscopy slides for immunofluorescence analysis. Primary antibodies used were human nuclear antigen (HNA; Millipore, 1:50) neuronal nuclei (NeuN; Millipore, 1:500), oligodendrocyte lineage transcription factor 2 (Olig2; Genetex, 1:250), and glial fibrillary acidic protein (GFAP; Abcam, 1:500). Details can be found in Supplementary Methods.

### Immunohistochemistry of Endogenous Tissues

Formalin-fixed whole coronal sections were routinely processed, embedded in paraffin, and stained with antibodies specific to NeuN (Millipore, 1:500), doublecortin (DCX; Abcam, 1:2000), GFAP (Biogenex, 1:4000), and Iba1 (Wako, 1:8000). Four healthy pigs of the same age that received no MCAO surgery, iNSC injection, or vehicle injection were sacrificed and brains were processed as described above to serve as normal control animals. Researchers were blinded to treatment group assignment when performing quantification analysis. Details can be found in Supplementary Methods.

### Gene Expression

Details can be found in Supplementary Methods.

### Statistics

All quantitative data was analyzed with SAS version 9.3 (Cary, NC) and statistical significances between groups were determined by two-way analysis of variance and post-hoc Tukey’s Pair-Wise comparisons. Treatments where p-values were ≤0.05 were considered to be significantly different.

### Data Availability

The datasets generated during the current study are available from the corresponding author on a reasonable request.

## Electronic supplementary material

Supplementary Methods
