# Supplementary material for: Induced Pluripotent Stem Cell-Derived Neural Stem Cell Therapy Enhances Recovery in an Ischemic Stroke Pig Model
Source: Sci Rep. 2017 Aug 30;7:10075. doi: 10.1038/s41598-017-10406-x (PMC5577218; doi:10.1038/s41598-017-10406-x)
Supplement: Supplementary file 1 — Supplementary Methods [file 41598_2017_10406_MOESM1_ESM.pdf]

Supplementary information for:

Induced Pluripotent Stem Cell-Derived Neural Stem Cell Therapy Enhances Recovery in an Ischemic Stroke Pig Model

Emily W. Baker[1,2], Simon R. Platt[1,3], Vivian W. Lau[1,2,3], Harrison E. Grace[1,2], Shannon P. Holmes[1,4], Liya Wang[5], Kylee Jo Duberstein[1,2], Elizabeth W. Howerth[1,6], Holly A. Kinder[1,2], Steve L. Stice[1,2], David C. Hess[7], Hui Mao[5], and Franklin D. West[1,2]

[1]Regenerative Bioscience Center, [2]Department of Animal and Dairy Science, [3]Department of Small Animal Medicine and Surgery, [4]Department of Veterinary Biosciences & Diagnostic Imaging, University of Georgia, Athens, GA 30602, USA; [5]Department of Radiology and Imaging Sciences, Emory University, Atlanta, GA 30329; [6]Department of Pathology, University of Georgia, Athens, GA 30602, USA; [7]Department of Neurology, Augusta University, Augusta, GA 30912

Corresponding author:
Franklin D. West
Regenerative Bioscience Center, Department of Animal and Dairy Science
University of Georgia, Rhodes Center for Animal and Dairy Science, 425 River Road, Athens, GA 30602-2771, USA
Phone: (706) 542-0988
Fax: (706) 542-7925
Email: westf@uga.edu

Supplementary Methods

Human Induced Pluripotent Stem Cell-Derived Neural Stem Cells (iNSC)

iNSC culture iNSCs were maintained on Matrigel-coated (BD Bioscience) tissue culture plates and maintained in neural stem cell media composed of Neurobasal medium (Thermo Fisher Scientific), 2% B-27 Supplement (Thermo Fisher Scientific), 1% non-essential amino acids (Life Technologies) 2 mM L-glutamine (Life Technologies), 1% penicillin/streptomycin (Invitrogen), and 20 ng/mL bFGF (R&D Systems). A complete media change was performed every other day. When iNSCs reached confluence, cells were enzymatically passaged using Accutase (Innovative Cell Technologies) and removed from the dish using a cell scraper. After passaging, the cells were re-plated at a ratio of 1:4.

iNSC differentiation iNSCs underwent spontaneous differentiation by culturing cells in neural stem cell media without bFGF for 2 and 4 weeks. Differentiation cultures were maintained on Matrigel-coated glass four-well chamber slides. Differentiated iNSC cultures underwent immunocytochemistry for mature neural cell marker detection.

iNSC flow cytometry and immunocytochemistry For both flow cytometry and immunocytochemistry, iNSCs were fixed with 4% paraformaldehyde (PFA; Electron Microscopy Sciences) for 15 minutes. Cells were permeabilized in blocking solution containing 0.1% Triton X-100, 1% Polyvinylpyrrolidone (PVP; Sigma-Aldrich), and 3% serum. Primary antibodies were diluted in blocking solution and incubated for one hour at room temperature. Primary antibodies used were Nestin (Neuromics, 1:200), Sox1 (R&D Systems, 1:20), Tuj1 (Neuromics, 1:200), MAP2 (Millipore, 1:500), GFAP (Abcam, 1:500), and Olig2 (Genetex, 1:250). Primary antibodies were detected using a fluorescently conjugated secondary antibody, Alexa Fluor (Invitrogen; 1:1000) and incubated for one hour at room temperature before

washing. For immunocytochemistry, stained slides were mounted with Prolong Gold with DAPI (Life Technologies) and imaged on an IX81 microscope with Disc-Spinning Unit (Olympus, Inc.) using Slide Book Software (Intelligent Imaging Innovations). For flow cytometry, cells were analyzed using a CyAn ADP flow cytometer (Beckman Coulter, Hialeah, Florida) and FlowJo Cytometry analysis software (Tree Star).

Labeling of iNSCs

iNSCs were labeled with 1,1′-dioctadecyl-3,3,3′,3′-tetramethylindotricarbocyanine iodide (DiR) at a concentration of 2 μg/mL diluted in phosphate buffered saline (PBS). After incubation at 37°C for 5 minutes, iNSCs were washed twice with PBS via centrifugation at 1500rpm. Cells were then resuspended in PBS at a final concentration 150,000 cells/μL for transplantation.

Pig Stroke Model

Animal Preparation Pigs were individually housed and fed a nutritionally complete diet. Room temperature was maintained at 26°C with a 12 h light/dark cycle.All animals were administered antibiotics daily for 3 days prior to surgery (Naxcel®; 4mg/kg IM). Pre-induction analgesia and sedation was obtained using xylazine (5 mg/kg IM), butorphanol (0.2 mg/kg IM), and midazolam (0.2 mg/kg IM). Anesthesia was induced with intravenous propofol to effect and 0.5-1.0 mL of 2% lidocaine was topically applied to the laryngeal folds to facilitate intubation and was maintained with 1.5% inhaled isoflurane (Abbott Laboratories) in oxygen. Artificial ventilation was utilized at a rate of 8-12 breaths per minute with a tidal volume of 5-10 mL/kg. During surgery, lactated ringers solution was administered intravenously in the ear vein at a rate of 5 mL/kg/hour. A Doppler probe placed on the ventral tail artery monitored heart rate continuously

throughout surgery, and a digital thermometer was used to check rectal temperature every 15 minutes. While the pig was positioned in sternal recumbency, the head was angled 45 degrees to its left with the mouth in an open position and secured to facilitate a right sided craniectomy approach. This position utilizes gravity to pull the cerebrum away from the skull during intracranial surgery. Hair between the eye and ear was shaved and the skin was prepared in a routine manner for sterile surgery using alternating applications of Betadine and 70% alcohol. The surgical site was draped in a standard fashion.

Middle Cerebral Artery Occlusion (MCAO) As previously described [1], injury was induced by performing a frontotemporal craniectomy with orbital rim ostectomy and zygomatic arch resection. A curvilinear skin incision was performed that extended from slightly above the right orbit to an area rostral to the ear canal. The temporal fascia and muscle were incised and reflected along with the skin flap. The branches of the superficial temporal artery and the associated vein were exposed and occluded using high frequency bipolar cautery forceps. Next, the insertion of the temporalis muscle was incised from below the zygomaytic arch and elevated away from the parietal bone. The periosteum overlying the zygomatic arch was incised and bluntly elevated away. The zygomatic arch was partially resected, which exposed the ventral aspect of the calvaria to the level of the orbital fissure. A surgical defect was generated in the exposed calvaria using a pneumatic drill and burr and extended using Kerrison rongeurs; the lateral portion of the roof of the orbit was rongeured away while the orbital contents were protected with a hand held surgical retractor. The visible dura mater was incised and reflected dorsally as a flap. The middle cerebral artery (MCA) was located just distal to the Circle of Willis at its origin and was permanently occluded utilizing the bipolar electrocautery forceps.

After occlusion, the exposed portion of the brain was covered with a sterile biograft made of porcine small intestine submuscosa (MatriStem, ACell). The temporalis muscle was routinely reattached and the skin was routinely re-apposed. Analgesia (Banamine®, 2 mg/kg IM) and antibiotic (Naxcel®, 4 mg/kg IM) was administered for 3 days postoperatively. Anesthesia was discontinued and pigs were returned to their pens upon extubation and monitored every 4 hours for the next 12 hours. Heart rate, respiratory rate, and temperature were recorded at each time point.

Magnetic Resonance Imaging

T1-weighted, DWI, and DWI-derived apparent diffusion coefficent (ADC) maps were analyzed using Osirix software. For brain tissue volumetric analysis, T1-weighted images were utilized to measure the areas of the ipsilateral (injured) and contralateral (normal) hemispheres. Hemisphere area and slice thickness were combined to obtain total hemisphere volume, and changes in the volume of the ipsilateral hemisphere were expressed as a percentage change relative to the contralateral hemisphere. Fractional anisotropy (FA), a measurement for white matter integrity, was calculated using DTI data in 3 different areas of the peri-infarct region while 3 comparable anatomical regions on the contralateral hemisphere were used as a control. The average change in FA at the area of the infarct was calculated relative to each respective area on the contralateral hemisphere. MRS was utilized to assess the level of selected neurometabolites in 3 separate regions in the peri-infarct area while 3 separate measurements were taken at a comparable area on the contralateral hemisphere. The integral of each peak was obtained from the magnetic resonance spectra for each neurometabolite of interest, and the change in peak integral of the ipsilateral region relative to the contralateral region was calculated. Mean transit time, time to

peak, and cerebral blood volume in the peri-infarct area and at a comparable region on the contralateral hemisphere were calculated from PWI. The change of cerebral perfusion was expressed as a percentage change in the ipsilateral hemisphere relative to the comparable area on the contralateral hemisphere.

iNSC Transplantation

Immediately prior to injection, iNSCs suspended in PBS were loaded into a glass micro-pipette syringe attached to a specialized 24 gauge needle (Hamilton Co, Reno, NV) and attached to the stereotaxic apparatus. Two separate injections of 5.0 x 10^6 iNSCs or vehicle only (n=4 per treatment group) were transplanted into two sites of the peri-lesional region at a depth of 6mm, which was defined as the interface between the cortex and subcortical white matter, at a rate of 2 μL/minute to prevent backflow. The location of the peri-lesional region was determined by 24hr post-stroke MRI scans of each animal. After transplantation was complete, the needle was retracted at a rate of 1 mm/minute to prevent backflow. Anesthesia was discontinued and the pigs were returned to their pens upon extubation.

Brain Tissue Collection and Processing

For iNSC transplantation analysis, brain sections were imaged using an IVIS Lumina II System (PerkinElmer, Waltham, MA). DiR fluorescence was visualized using an excitation wavelength of 745 nm and emission wavelength set to Indocyanine Green filter. Tissue areas of high DiR fluorescence were collected, fixed in 4% PFA (Electron Microscopy Sciences), cryoprotected in 30% sucrose, embedded in Tissue-Tek OCT compound (Sakura), and stored at -80°C. For gene expression, brain tissue samples were collected from the peri-lesion area, dissected to separate

gray and white matter, snap frozen in liquid nitrogen, and stored at -80°C. For tissue immunohistochemical analysis, whole representative sections at the level of the infarct were immersed in 10% buffered formalin.

Immunofluorescence of iNSC Transplantation Sites

After rehydration with PBS, tissues were blocked in 10% normal donkey serum and 0.3% Triton X-100 for one hour. Primary and secondary antibodies were diluted in PBS with 1% normal donkey serum and 0.3% Triton X-100. Primary antibodies were incubated overnight at 4°C and were detected using a species-specific fluorescently conjugated secondary antibody by Alexa Fluor (Invitrogen; 1:1000 for HNA, NeuN, and Olig2, 1:500 for GFAP) which was incubated for one hour at room temperature. Slides were mounted in Prolong Gold with DAPI (Invitrogen) and allowed to dry overnight before microscopic inspection. Imaging was performed on a IX81 microscope with Disc-Spinning Unit (Olympus, Inc.) utilizing the Z-stack capabilities of Slide Book Software (Intellegent Imaging Innovations). Quantification was performed by imaging 5 random fields for each animal and antibody and utilizing the cell counter plugin on ImageJ 2.0 software [2].

Immunohistochemistry of Endogenous Tissues

Heat induced antigen retrieval was performed for all antibodies using citrate buffer at pH6 (DAKO). Detection was performed utilizing biotinylated antibodies and a streptavidin label (4plus Streptavidin HRP, Biocare). A HRP label and DAB chromagen (DAKO) was used and all sections were lightly counterstained with hematoxylin. Microscopic inspection and imaging was performed on a Nikon Eclipse TE300 inverted microscope. All quantification was carried out on

ImageJ 2.0 software. For NeuN, GFAP, and Iba1, images were taken at the lesion border ranging from the ectomarginal gyrus to the piriform lobe. For quantification of NeuN, positive cells were manually counted using the cell counter plugin on ImageJ 2.0 and expressed as cells/mm^2 [2]. For semi-quantitative analysis of GFAP and Iba1, the total area of immunoreactivity corresponding to increased optical density was determined by ImageJ software. For DCX, as described in previous studies [3], DCX+ neuroblasts in three separate anatomical regions were analyzed at the level of the caudate nucleus: 1) the neuroblasts positioned directly adjacent to the ependymal cells lining the the lateral ventricle (ventricular subventricular zone; vSVZ), 2) the neuroblasts organized laterally into chains adjacent to but distinct from the vSVZ (abventricular SVZ; aSVZ), and 3) the neuroblasts that have migrated laterally to the lesion border. DCX+ cells were manually counted using the cell counter plugin on ImageJ and expressed as cells/mm^2.

Gene Expression

Brain tissue samples allocated for gene expression analysis were weighed, and approximately 150mg of tissue was transferred to cold RNAlater-ICE (Thermo Fisher Scientific) and allowed to thaw overnight at -20°C. Total RNA was extracted using an RNeasy mini kit (Qiagen) according to the manufacturer's protocol. The quality and quantity of the isolated RNA was measured on a NanoDrop 8000 (Thermo Fisher Scientific). Nine hundred nanograms of RNA from each sample was used for reverse transcription using the High Capacity cDNA Reverse Transcription Kit (Thermo Fisher Scientific) according to the manufacturer's instructions. Quantitative PCR was performed using a customized TaqMan Array Card (Thermo Fisher Scientific) on an Applied Biosystems 7900HT Real-Time PCR System (Thermo Fisher Scientific) using SDS v2.4 software. Data was processed using ExpressionSuite Software v1.0.3 to obtain C_T values. $\Delta\Delta C_T$

and Relative Quantification values were calculated manually using Sus scrofa-specific GAPDH as a housekeeping control, and data was expressed as a fold change in iNSC treated samples relative to non-treated samples.

Supplementary References

1. Platt, S. R. *et al.* Development and characterization of a Yucatan miniature biomedical Pig permanent middle cerebral artery occlusion stroke model. *Experimental & translational stroke medicine* **6**, 5, doi:10.1186/2040-7378-6-5 (2014).
2. Schneider, C. A., Rasband, W. S. & Eliceiri, K. W. NIH Image to ImageJ: 25 years of image analysis. *Nat Methods* **9**, 671-675 (2012).
3. Costine, B. A. *et al.* The subventricular zone in the immature piglet brain: anatomy and exodus of neuroblasts into white matter after traumatic brain injury. *Developmental neuroscience* **37**, 115-130, doi:10.1159/000369091 (2015).
